# Assessing the Potential of *Aconitum Laeve* Extract for Biogenic Silver and Gold Nanoparticle Synthesis and Their Biological and Catalytic Applications

**DOI:** 10.3390/molecules29112640

**Published:** 2024-06-03

**Authors:** Shahbaz Ahmad, Qianqian Xu, Muhammad Tariq, Meijie Song, Chao Liu, Hai Yan

**Affiliations:** 1 School of Chemistry and Biological Engineering, University of Science and Technology Beijing, Beijing 100083, China; shahbazbbt19@yahoo.com (S.A.); meijie_song@126.com (M.S.); b20190371@xs.ustb.edu.cn (C.L.); 2 State Key Laboratory of Chemical Resource Engineering, Beijing University of Chemical Technology, Beijing 100029, China; muhammadtariq.apex@gmail.com

**Keywords:** green synthesis, *Aconitum Laeve*, Ag and Au nanoparticles, biological assays, photocatalysis

## Abstract

The adoption of green chemistry protocols in nanoparticle (NP) synthesis has exhibited substantial potential and is presently a central focus in research for generating versatile NPs applicable across a broad spectrum of applications. In this scientific contribution, we, for the first time, examined the ability of *Aconitum Laeve* (*A. Laeve*) crude extract to synthesize silver and gold nanoparticles (AgNPs@AL; AuNP@AL) and explored their potential applications in biological activities and the catalytic degradation of environmental pollutants. The synthesized NPs exhibited a distinctive surface plasmon resonance pattern, a spherical morphology with approximate sizes of 5–10 nm (TEM imaging), a crystalline architecture (XRD analysis), and potential functional groups identified by FTIR spectroscopy. The antibacterial activity was demonstrated by inhibition zones that measured 16 and 14 mm for the AgNPs@AL and AuNP@AL at a concentration of 80 µg/mL against *Staphylococcus aureus* and 14 and 12 mm against *Escherichia coli*, respectively. The antioxidant potential of the synthesized NPs was evaluated using 2,2-diphenyl-1-picrylhydrazyl (DPPH), 2-Phenyl-4,4,5,5-tetramethylimidazoline-1-oxyl 3-Oxide (PTIO), and 3-ethylbenzothiazoline-6-sulfonic acid (ABTS) assays. Our findings suggest that the AuNP@AL effectively countered the tested radicals considerably, displaying IC_50_ values of 115.9, 103.54, and 180.85 µg/mL against DPPH, PTIO, and ABTS, respectively. In contrast, the AgNPs@AL showed IC_50_ values of 144.9, 116.36, and 95.39 µg/mL against the respective radicals. In addition, both the NPs presented significant effectiveness in the photocatalytic degradation of methylene blue and rhodamine B. The overall observations indicate that *A. Laeve* possesses a robust capability to synthesize spherical nanoparticles, exhibiting excellent dispersion and showcasing potential applications in both biological activities and environmental remediation.

## 1. Introduction

Nanobiotechnology is a rapidly progressing domain dedicated to developing and optimizing nanoscale materials for diverse applications across a wide range of scientific disciplines [1]. These nanostructures exhibit distinctive characteristics stemming from their exceptionally small sizes and expanded surface areas compared to larger or bulk masses [2]. In the field of nanoscience, metal-based nanoparticles (NPs) of silver (Ag) and gold (Au) are particularly important due to their diverse nature, enhanced catalytic behavior, usage in pharmaceuticals, gene expression, cosmetics, and household consumables [3,4,5,6,7]. Utilizing their strong antimicrobial properties, AgNPs are widely applied in the production of food processing items, topical ointments, and medical implants [8,9,10], particularly effective alternatives for addressing emerging microbial resistance. AgNPs exhibit potent bactericidal activity at concentrations that do not induce cytotoxicity in human cells [11]. Furthermore, they synergistically enhance conventional antibiotics’ antibacterial effectiveness, even when confronted with multi-resistant bacteria [12,13]. The antimicrobial action of AgNPs entails a complex process, including inhibiting metabolically crucial enzymes, scavenging reactive oxygen species (ROS), disrupting microbial membranes, and interfering with DNA replication [14,15,16,17].

Similarly, AuNPs have gained substantial recognition as antibacterial agents due to their biocompatibility and low cytotoxicity [18,19,20]. Some of the major characteristics of AuNPs include their diameters being slightly larger than drug efflux pumps, their adaptable valence states, and their capacity to interfere with intra-protein interactions [20,21,22]. Moreover, they can be functionalized with various bioactive agents, showcasing robust synergistic effects that significantly enhance the antibacterial efficacy of conventional antibiotics, even against multidrug-resistant bacteria. [23,24]. This capability allows them to enhance their inherent antibacterial activity through synergistic effects [25]. Moreover, the plasmonic attributes of AuNPs have showcased significant potential in both therapeutic and diagnostic applications. These applications encompass phototherapy, thermal ablation, cell imaging, and targeted drug delivery [26,27]. It is currently premature to determine whether NPs of Ag and Au could augment antibiotic efficacy in medicine or potentially replace them entirely in treating systemic and local infections. Nevertheless, exploring potential advancements in synthesizing such NPs and their application in the treatment of multiple bacterial strains is necessary.

Besides this, biogenic Ag and AuNPs possess the capability to scavenge the overproduction of ROS, thus demonstrating potential effectiveness in mitigating oxidative damage to human cells [28,29,30]. Oxidants trigger molecular-level oxidative modifications in biological systems, leading to damage and ultimately expediting cellular death [31]. This phenomenon arises when oxidative substances are excessively generated or accumulated, and defense mechanisms prove inadequate. Biogenically synthesized NPs have been documented for their radical scavenging capabilities [31,32,33], attributed to the simultaneous activity of polyphenols (capping agents) as antioxidant agents and AgNPs functioning as catalysts. The existence of polyphenolic compounds within bioinspired AgNPs functions as a singlet oxygen quencher and hydrogen donor, thereby providing a defensive mechanism against oxidative stress [34].

Beyond their biological applications, both Ag and AuNPs have also exhibited promising potential in catalyzing the conversion of environmental toxic pollutants. Hence, they hold significant importance in environmental remediation [35,36,37]. These NPs have been acknowledged for their capacity to promote the degradation of various dyes, which can otherwise be harmful to biological samples and have adverse effects on human health [38]. Considering the biomedical and catalytic potentials of Ag and AuNPs, we were motivated to design biogenic NPs of these metals and explore their capabilities across various disciplines. Aligned with the mentioned applications, there is an immediate requirement for a straightforward and efficient method to synthesize metal nanoparticles with advantageous properties, aiming to augment their performance in both bioactive and catalytic domains.

Most chemical procedures employed in synthesizing metal NPs often involve using toxic organic solvents and reducing molecules, presenting potential risks to the environment and biological systems. In contrast, physical methods demand advanced equipment and rigorous conditions and are restricted by low production rates and high energy utilization, leading to increased costs [25]. Biological approaches have become increasingly popular for their ability to generate cost-effective and sustainable alternatives to NPs. The production of NPs through green procedures provides minimal toxicity to the environment and life on earth, as opposed to traditional preparation methods [39,40]. The plant-mediated green synthetic approach is devoid of using bacteria or fungal cultures and avoids potential hazards to living beings and the environment [41,42].

Species belonging to the genus *Aconitum Laeve* (*A. Laeve*) have demonstrated a wide range of pharmacological activities, especially cardiotonic, anti-inflammatory, and analgesic potential [43,44]. The plant *A. Laeve* is well known for the presence of potent diterpenoid alkaloids that exhibit promising antibacterial, antiviral, and antioxidant activities [45,46,47]. The potential of *A. Laeve* to mediate the reduction of Ag into NPs has not been explored previously. In this study, we present, for the first time, the green synthesis of Ag and AuNPs utilizing the methanol crude extract of *A. Laeve* as a source of phytochemicals for the reduction and stabilization of AgNPs and AuNPs. The prepared NPs were subsequently utilized as nanomedicine against specific bacterial strains and as a catalyst in antioxidant assays and in environmental remediation.

## 2. Results and Discussion

### 2.1. Characterization of Ag and Au NPs

#### 2.1.1. UV–Visible Spectroscopic Examination

The commencement of NP synthesis can be visually evident through a distinct color alteration when the plant extract is introduced to the predetermined concentration of the precursor solution. NPs within the solution absorb light at a particular wavelength and then reflect it at others, thereby manifesting a unique color that signals the formation of the desired material. In our experiment, the incorporation of *A. Laeve* extract into the predefined Au precursor solution led to a gradual deepening of a violet color. This observation suggests the reduction of Au ions in the solution and subsequent nucleation, facilitating the formation of the desired nanoparticles. Likewise, it was noted that adding the plant extract to the designated concentration of the Ag precursor led to the emergence of a dark color that grew more pronounced with time. This observation suggests that the Ag ions in the solution experienced reduction and subsequent nucleation, ultimately forming the desired nanoparticles. This alteration in color can be conveniently tracked by examining the surface plasmon resonance (SPR) pattern of the materials using UV–Vis spectroscopy. Both Ag and AuNPs display UV bands within the visible spectrum, with AgNPs typically ranging from 400 to 470 nm and the AuNPs from 500 to 580 nm [48,49,50]. Many parameters, such as the size, structure, and dielectric properties of the reaction medium used for producing the resulting nanoparticles, affect the material’s surface photo response (SPR) pattern.

**Figure 1:**
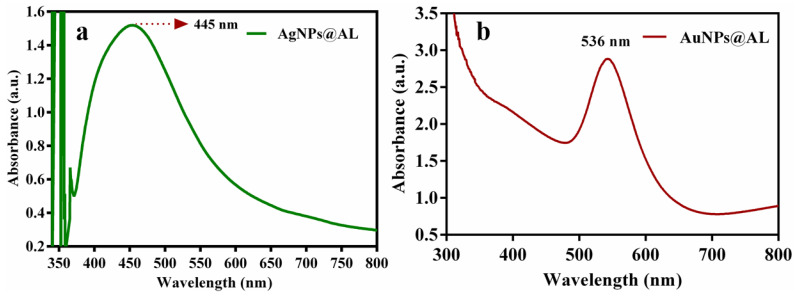
UV–visible spectroscopic analysis of (**a**) AgNP@AL and (**b**) AuNP@AL synthesized using *A. Laeve* crude extract.

In our investigation, the synthesized AgNP@AL revealed an SPR peak at 445 nm (Figure 1a), validating the successful formation of the intended material, consistent with published reports [51,52]. A continual increase in the SPR intensity was noted over time, signifying ongoing Ag reduction and nanoparticle synthesis. The stability of the SPR peak was established after 2 h, indicating the completion of the reaction within this timeframe. It has been previously established that NPs exhibiting a spherical morphology typically manifest an SPR frequency in the range of 405–475 nm [53,54]. Our results indicate that the fabricated AgNPs are spherical, a conclusion further validated through TEM investigation. Similarly, the bio-fabricated AuNP@AL exhibited an SPR peak at 536 nm (Figure 1b), confirming the successful formation of the nanomaterial, consistent with findings in published reports [55,56]. In summary, the comprehensive observations from visual and UV–visible analyses indicate that the phytochemicals in the *A. Laeve* extract efficiently reduce Ag and Au ions and act as efficient stabilizing biomolecules in the fabrication of Ag and AuNPs.

#### 2.1.2. XRD Analysis of the Prepared NPs@AL

**Figure 2:**
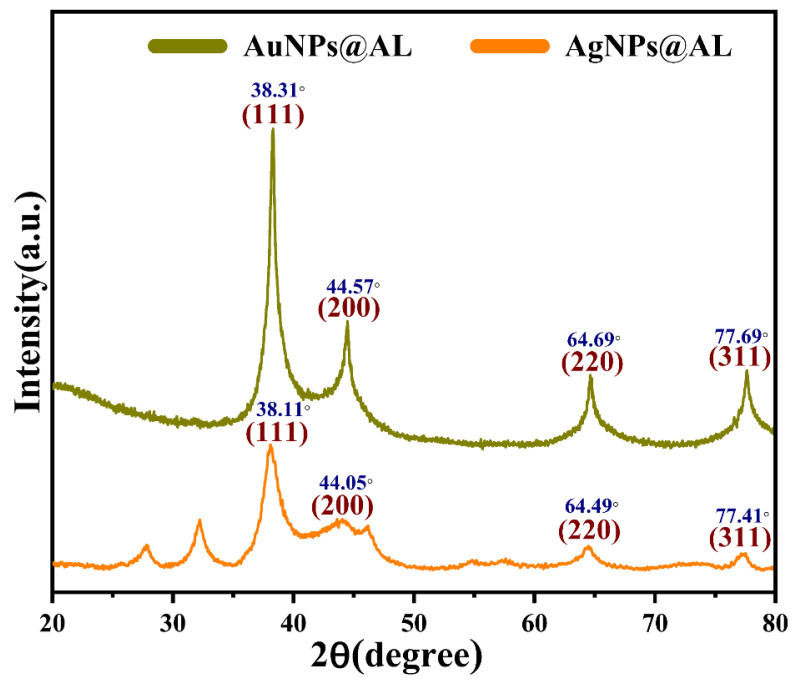
X-ray diffraction (XRD) pattern of Ag and Au NPs synthesized using *A. Laeve* crude extract, displaying prominent diffraction peaks characteristic of crystalline Ag and Au.

We conducted an XRD assessment to determine if the biosynthesized NPs@AL are crystalline or amorphous, and the findings are presented in Figure 2. The face-centered cubic structural characteristics of the synthesized AgNP@AL were demonstrated by the peak positions at 2-theta 38.11, 44.05, 64.49, and 77.41, which matched the typical Bragg’s reflections for metallic Ag [48,57]. There were extra unidentified peaks in the collected data, implying the potential presence of bio-organic phase crystals [58]. Similarly, the AuNP@AL fabricated in this study disclosed features representative of a face-centered cubic structure, as evidenced by peak positions at 2-theta values of 38.310, 44.570, 64.690, and 77.690. These peaks agree with the characteristic Bragg’s reflections for crystalline Au [35]. XRD analysis further confirms the crystalline nature of the AuNPs produced by the crude extract of *A. Laeve*. The presence of diverse reducing biomolecules in the extract contributes to the stabilization of both the Ag and AuNP@AL and is responsible for imparting the crystalline structure to the NPs@AL, a phenomenon extensively studied in various biosynthesized nanoparticles [59,60].

#### 2.1.3. Morphological Analysis

**Figure 3:**
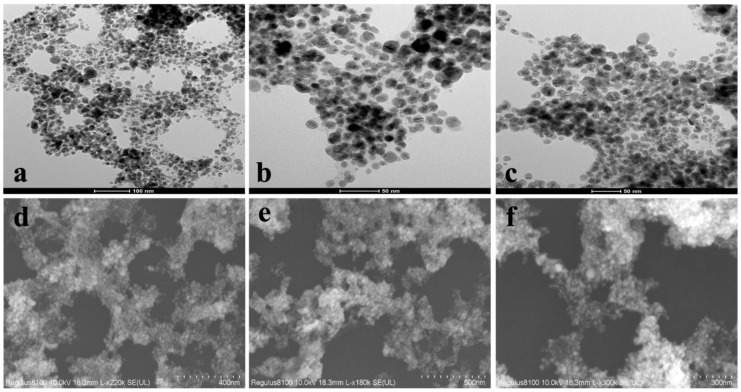
TEM images captured at various magnifications illustrating particle size and dispersion (**a**–**c**). SEM micrographs at different magnifications (**d**–**f**).

The morphology, size, and dispersion of the prepared nanoparticles were examined through SEM and TEM analysis. The prepared AgNP@AL showcased an approximately spherical morphology and were well separated from one another, signifying successful capping and the absence of aggregation as observed in TEM images (Figure 3a–c). A distinct layer was observed surrounding the synthesized NPs, and this thin layer can be attributed to the phytochemicals acting as capping agents. This layer aids in preventing the aggregation process. The existence of such an organic layer enveloping metal-based nanomaterials has also been documented in previous studies [58,61,62]. The generation of evenly dispersed NPs implies that the *A. Laeve* crude extract encompasses active phytochemicals that can proficiently reduce and subsequently cap the Ag ions into the AgNP@AL. Subsequent SEM analysis was conducted to visualize the morphology and distribution of the synthesized NPs. The observations revealed a spherical shape with well-spaced particles, indicative of the effective capping and stabilization achieved through the phytochemicals present in the methanol crude extract of *A. Laeve* (Figure 3d–f).

**Figure 4:**
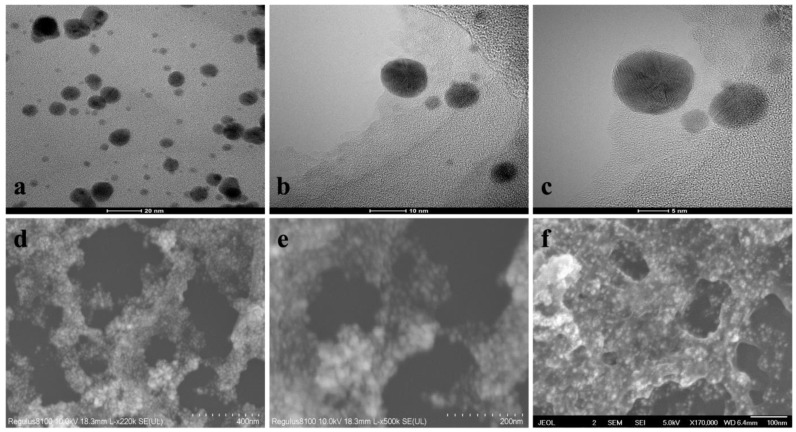
(**a**–**c**) TEM micrographs of the prepared AuNP@AL at different magnifications and (**d**–**f**) SEM images obtained at different magnifications.

Similarly, TEM observations revealed that the AuNP@AL exhibited approximately spherical shapes with sizes around 5–10 nm and excellent dispersion in the colloidal suspension. Aggregation was not observed, indicating a strong stabilizing influence of the biomolecules in the *A. Laeve* extract (Figure 4a–c). The equivalent SEM pictures of the *A. Laeve*-induced AuNPs showed an identical configuration of NP shape, size, and distribution (Figure 4d,e). It is also important to note that the AuNP@AL are covered in a thin layer, which adds more evidence that phytochemicals play a role in the formation of AuNPs [57].

#### 2.1.4. Dynamic Light Scattering Study

**Figure 5:**
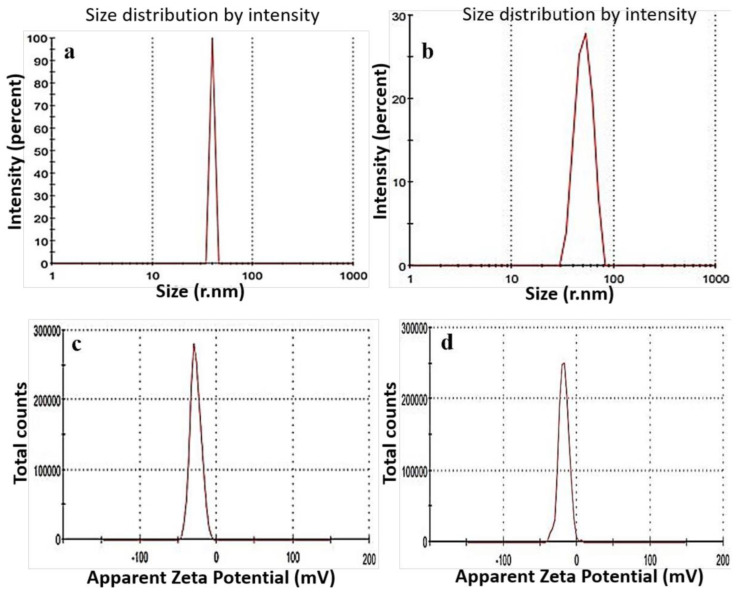
DLS measurements of the prepared NPs@AL. (**a**) Size distribution of the AgNP@AL and (**b**) AuNP@AL. (**c**,**d**) Zeta potential values of the AgNP@AL and AuNP@AL.

The size distribution of the fabricated NPs was examined using the dynamic light scattering (DLS) technique. DLS is a crucial analytical technique employed for determining the size distribution, polydispersity index, and zeta potential values of nanomaterials prepared in solution. We also utilized this technique to explore these distinctive features of the synthesized NPs@AL. The findings from DLS analysis indicated that the majority of the AgNP@AL were around 39 nm (Figure 5a) with a PDI value of 0.35. Similarly, the results obtained from DLS analysis revealed that the predominant size of the AuNP@AL was around 70 nm (Figure 5b), accompanied by a PDI value of 0.32. The particle sizes obtained from DLS measurements are considerably larger than those obtained from TEM investigations. The variations in particle sizes observed through DLS and TEM analyses can be credited to the distinctive principles and detection approaches employed by these two methodologies. In addition, compared to the results of TEM analysis, the DLS approach yields a wider size distribution since it analyzes the hydrodynamic diameter of particles, which is greatly influenced by water molecules [63]. The stability of NPs is crucial for their dispersion in solution and applications across various scientific domains. Zeta potential values are a useful indicator for evaluating this characteristic of NPs, reflecting the overall charge maintained by a sample in a given solution. The AgNP@AL had a zeta potential of −26 mV (Figure 5c), whereas the AuNP@AL exhibited a zeta potential of −25 mV (Figure 5d), indicating that biomolecules with negative charges surround these particles. This negativity fosters electrostatic repulsion among the NPs, potentially preventing their aggregation and improving their long-term stability. The TEM findings (Figure 4), which reveal substantial dispersion of particles with no evidence of aggregation, are in line with the findings of zeta potential investigation.

#### 2.1.5. EDX Analysis of the Prepared Ag and AuNPs

**Figure 6:**
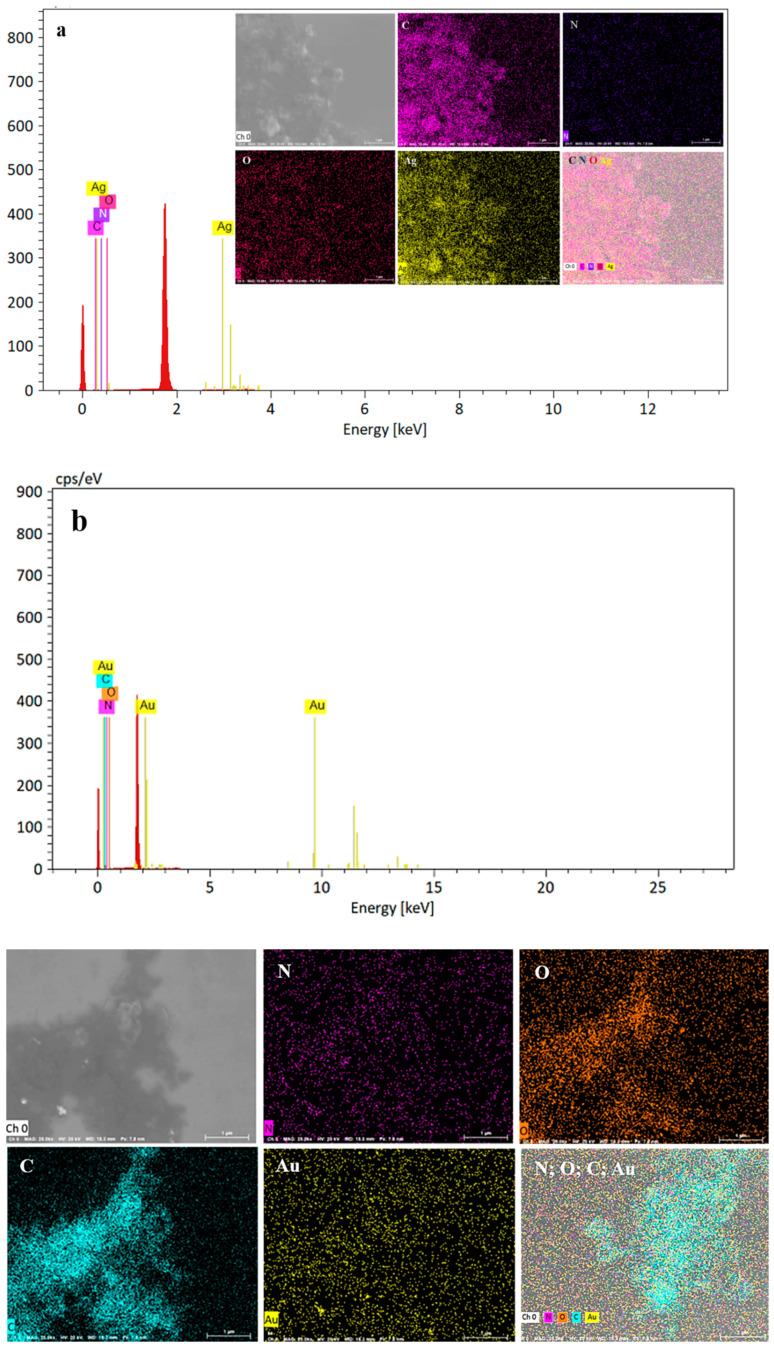
EDX spectral profile and the corresponding mapping of the (**a**) AgNP@AL and (**b**) AuNP@AL mediated by *A. Laeve* extract.

EDX is a reliable method for assessing the elemental composition of the produced material. Figure 6a depicts the EDX spectrum of the AgNP@AL synthesized through a green approach. The prominent peak around 3 eV signifies Ag and is linked to the presence of AgNPs [64,65]. Likewise, EDX mapping validates the existence of Ag in the bio-fabricated AgNPs (Figure 6a inset).

Similarly, the EDX pattern of the AuNP@AL was examined, revealing the presence of signals characteristic of Au, as shown in Figure 6b [66]. Furthermore, traces of carbon, oxygen, and nitrogen were identified in the EDX spectrums of both NPs, likely originating from the phytochemicals acting as capping agents in the synthesized NPs. Additionally, the occurrence of Au in the synthesized AuNP@AL was corroborated through EDX mapping analysis.

#### 2.1.6. FTIR Investigation of the Plant Extract and NPs@AL

**Figure 7:**
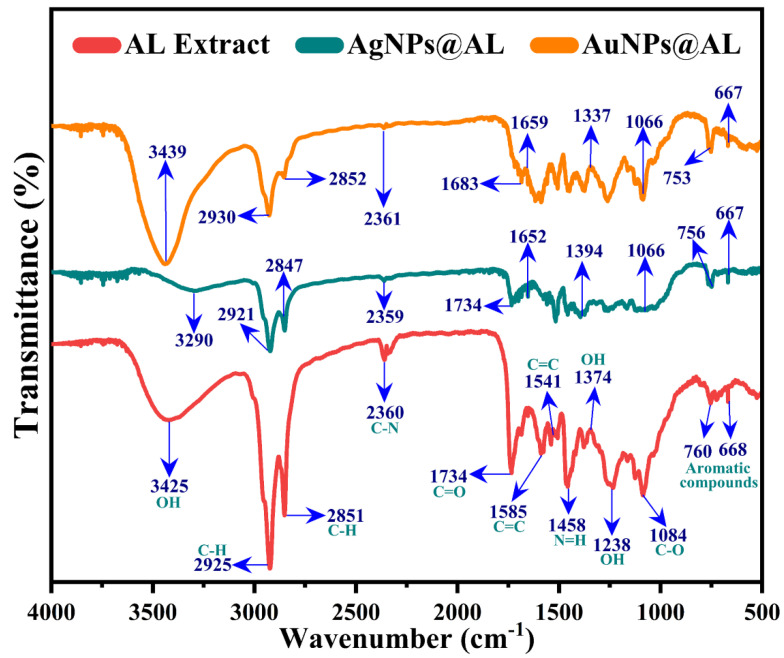
Comparative IR spectral analyses of *A. Laeve* extract and the corresponding NPs@AL. The spectral examination reveals a similar signature of functionalities in the *A. Laeve* crude extract, with minor shifts in band positions in the synthesized NPs@AL.

A comparative Fourier transform infrared (FTIR) analysis of the *A. Laeve* extract and the NPs@AL were performed to elucidate the functional groups participating in the reduction and stability of the synthesized NPs. As illustrated in Figure 7, both the *A. Laeve* extract and the synthesized NPs@AL present a comparable FTIR band pattern, featuring slight modifications in band positions and their corresponding intensities in the prepared NPs@AL. The predominant peaks identified in the IR spectrum of the *A. Laeve* crude extracts were located at 3320, 2927, 2852, 1734, 1590, 1448, 1270, 1083, and 760 cm^−1^. The spectral band at 3320 cm^−1^ is assigned to the OH stretching vibration of polyphenols, while the signals at 2927 cm^−1^ and 2852 cm^−1^ correspond to aliphatic CH stretch. The most prominent peak at 1734 cm^−1^ indicates the carboxylic C=O stretch of flavonoids present in the *A. laeve* crude extract. The vibration stretch observed at 1597–1520 cm^−1^ corresponds to the C=C stretch in the aromatic ring, confirming the presence of an aromatic group. The band at 1448 cm^−1^ may be attributed to the N-H stretching in amine, the 1083 cm^−1^ band signifies the C-O stretch of ether, and the 760 cm^−1^ band could originate from aromatic species [67,68,69,70,71,72]. Analysis of the FTIR spectra for both the AgNP@AL and AuNP@AL verifies the existence of functional groups similar to those in the plant extract, with slight shifts in their respective positions. These observations indicate that potential moieties, including amides, polyphenols, and carboxylic acids in the plant extract, could be responsible for the successful stabilization of the prepared nanoparticles.

#### 2.1.7. XPS Spectral Study

**Figure 8:**
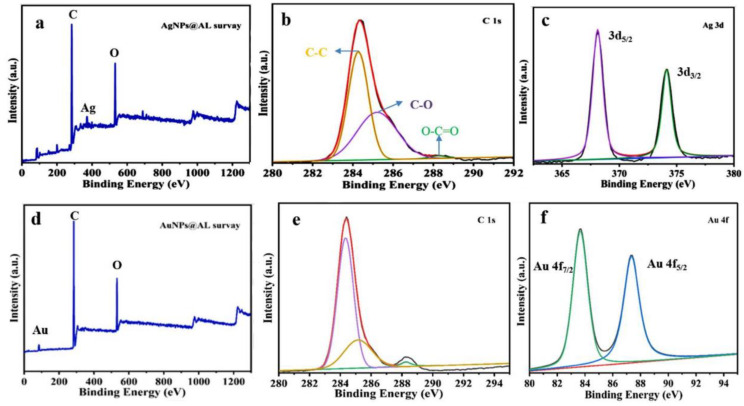
XPS spectral investigation of the prepared NPs@AL. (**a**) XPS survey, (**b**) deconvoluted spectra of C 1s, and (**c**) Ag 3d of the AgNPs@AL. (**d**) Presents the XPS survey, (**e**) deconvoluted spectra of C 1s, and (**f**) Au 4f of the AuNP@AL.

XPS is a crucial analytical technique employed not only for identifying the elements in a sample but also for determining their oxidation states. In Figure 8, the XPS spectrum of the AgNP@AL is displayed. As depicted in Figure 8a, the overall spectra of the AgNPs reveal a prominent peak corresponding to metallic Ag (366.8 eV) along with C at 284 eV and O at 531.5 eV. Similarly, the XPS spectra of the individual elements were then deconvoluted, with the results presented in Figure 8b,c. The observed peaks at 369 and 374 eV align with the Ag 3d5/2 and Ag 3d3/2 binding energies, respectively, signifying the distinctive signature of Ag in the zero oxidation state [73]. Similarly, the presence of three peaks at 284 eV, 285.3 eV, and 287.5 eV in the deconvoluted C 1s spectra may be associated with sp^2^ hybridization, sp^3^ hybridization, and O-C=O functionality, respectively [70,74]. Likewise, the overall survey of the AuNP@AL reveals corresponding signals of Au, C, and O at binding energies of 85, 285.5, and 531.4 eV, respectively (Figure 8d). The deconvolution of the C 1s signal reveals three bands at positions 284.3, 285.1, and 288.3 eV, signifying the respective linkages of C-C at 284.3 eV, C-OH at 288.1 eV, and O-C-O at 288.3 eV [71] (Figure 8e). Likewise, in the deconvoluted XPS spectra of the Au 4f region of the AuNP@AL, the signals at 83.6 and 87.3 eV align with Au 4f7/2 and Au 4f5/2 electrons, respectively [72] (Figure 8f). The identification of two prominent peaks in the 4f region distinctly indicates the presence of elemental gold (Au0) in the synthesized AuNP@AL [72]. Additionally, the identification of C-C, C-OH, and O-CO signals in the XPS analysis indicates an interaction between these functional groups from the *A. Laeve* extract and Au in the synthesized AuNPs. This interaction corresponds with the presence of these groups, as identified in the FTIR spectral analysis.

### 2.2. Biological Applications of Ag and AuNP@AL

#### 2.2.1. Antibacterial Potency of the Prepared NPs@AL

**Figure 9:**
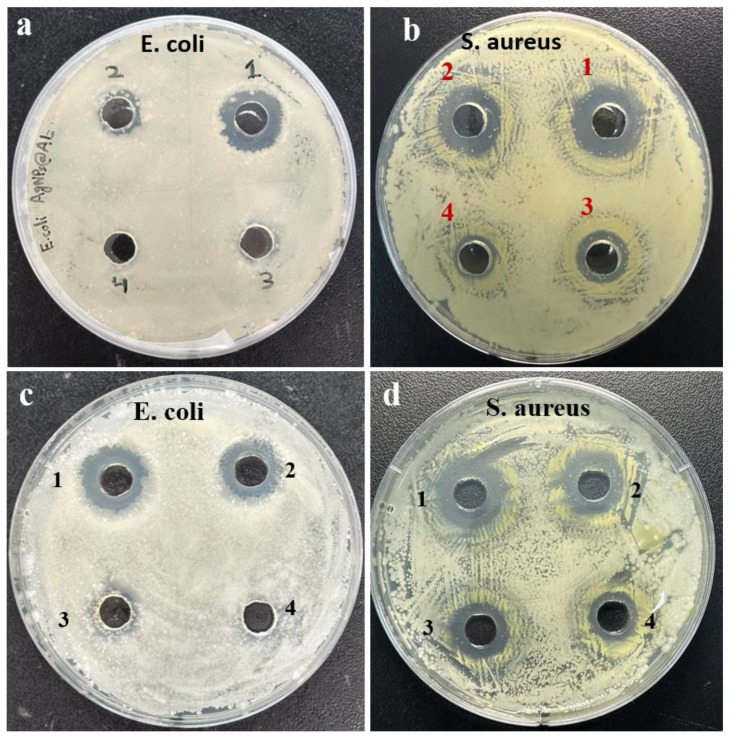
The antibacterial activity of the AgNP@AL against (**a**) *E. coli* and (**b**) *S. aureus*, and of the AuNP@AL (**c**,**d**), respectively, at different concentrations used (1 = 80 μg/mL, 2 = 40 μg/mL, 3 = 20 μg/mL, and 4 = 10 μg/mL).

The increasing bacterial resistance to antibiotics represents a significant global health challenge, underscoring the urgent need to develop novel and potent agents capable of treating a diverse range of microorganisms and effectively combating the escalating bacterial resistance. Metal-based nanomaterials, specifically noble metal NPs, have demonstrated considerable potential as antimicrobial agents due to their intrinsic antibacterial activities [75,76,77,78]. Therefore, substantial research interest has been focused on the development of Ag and AuNPs. Considering the antibacterial capabilities of Ag and AuNPs, we investigated the antibacterial effects of these NPs@AL against *E. coli*, a Gram-negative bacterium, and *B. subtilis*, a Gram-positive bacterium. Both the Ag and AuNP@AL demonstrated a prominent response against the examined pathogens, and the antibacterial activity is depicted in Figure 9. The most substantial inhibition zones, observed at the highest concentration of the AgNP@AL (80 µg/mL), were recorded at 12 mm and 16 mm against *E. coli* and *S. aureus* (Figure 9a and Figure 9b), respectively. Likewise, the AuNP@AL exhibited significant potential in inhibiting the experimental bacteria with inhibition zones measured at 14 mm and 16 mm against *E. coli* and *S. aureus* (Figure 9c and Figure 9d), respectively. Both the Ag and AuNP@AL demonstrated better antibacterial efficacy against *S. aureus* in comparison to *E. coli*, exhibiting a dose-dependent trend. Although both the AgNP@AL and AuNP@AL displayed almost identical antibacterial activities at the highest dosage administered, the AuNPs exhibited sustained efficacy at lower concentrations compared to the AgNP@AL. This heightened potency of the AuNPs at lower doses may be attributed to their smaller sizes and superior dispersion in solution. Studies have indicated that the size of nanomaterials plays a crucial role in bactericidal activity. Small nanoparticles (2–10 nm) are known to cause more membrane damage than larger ones due to their increased surface area contact with bacterial cells [78,79]. The diminished toxicity of the NPs@AL against *E. coli* can be ascribed to the diversity in cell wall composition between Gram-positive and Gram-negative bacteria. More precisely, the heightened negative charge observed in the *E. coli* cell wall, attributed to the existence of an outer lipid membrane, stands in contrast to the Gram-positive *S. aureus* [63]. As evidenced by the zeta potential (Figure 5), the prepared NPs@AL exhibit a negative charge. This negative charge results in increased electrostatic repulsion between the AgNP@AL and *E. coli* compared to *S. aureus*, consequently hindering the attachment and permeation of particles into the cell.

**Scheme 1:**
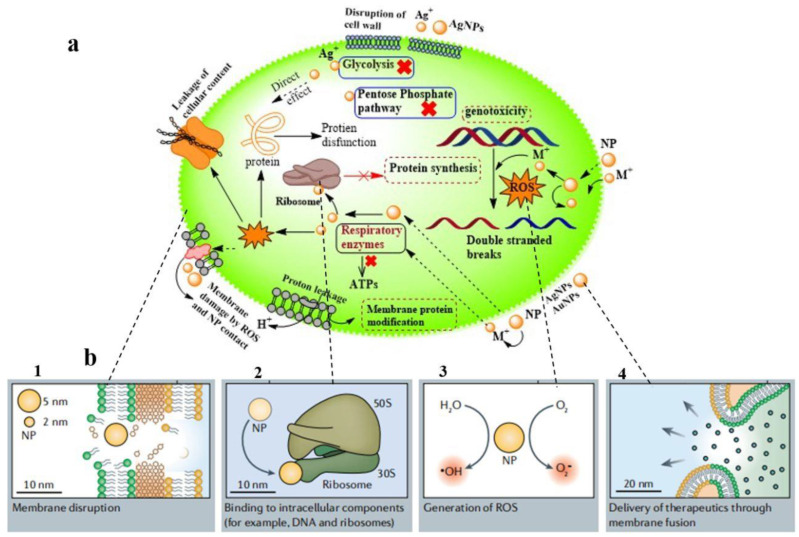
(**a**) Multiple modes of action by Ag+ and Au ions from Ag and AuNPs. NPs can bind to a range of intracellular components including ribosomes, proteins, and DNA, and generate ROS, disrupting their functions. Additionally, nanomaterials can serve as carriers for therapeutic agents, thereby facilitating the delivery of their cargo. (**b**) A schematic illustration shows: (1) membrane disruption upon contact with nanoparticles, (2) binding of nanoparticles with intracellular components, such as ribosomes, (3) the mechanism of hydroxyl radicals and superoxide ROS generation, and (4) the use of nanomaterials as carriers for therapeutic agents. Certain types of nanomaterials can easily penetrate bacterial cells through membrane fusion, thereby facilitating the delivery of their cargo.

The mechanisms underlying the activity of Ag and AuNPs on bacteria have not been fully elucidated. However, when attached to the bacterial surface, these NPs can induce damage and alter the membrane potential, leading to cytoplasmic leakage [63,80]. The findings also suggest that Ag and AuNPs can interact with bacterial DNA, resulting in its leakage and exerting genotoxic effects. The antibacterial activity of AuNPs often relies on the associated compounds and surface ligands. Moreover, AuNPs can bind with bacterial ribosomes/chromosomes, thereby inhibiting protein synthesis [81,82]. AgNPs have inherent antibacterial properties due to their ability to release silver ions. Subsequently, AgNPs are comparatively more antibacterial than AuNPs. In addition, other parameters such as surface chemistry, shape and size of the AgNPs and AuNPs also influence their antibacterial activities [83]. Furthermore, Ag- and Au-based nanomaterials have been documented to stimulate the generation of intracellular reactive oxygen species (ROS), which can induce cell damage through various mechanisms, encompassing both cytotoxic and genotoxic effects [84,85]. The Ag ions released from the attached NPs can hinder the function of enzymes by displacing structurally crucial metals from metal-containing enzymes, also known as metalloenzymes [86]. Therefore, the hypothesis posits that metal particles may concurrently utilize multiple mechanisms to eliminate microbes, thereby heightening the difficulty for bacteria and other pathogens to develop resistance to metal-based nanomedicines (Scheme 1).

#### 2.2.2. DPPH Radical Scavenging Activity

**Figure 10:**
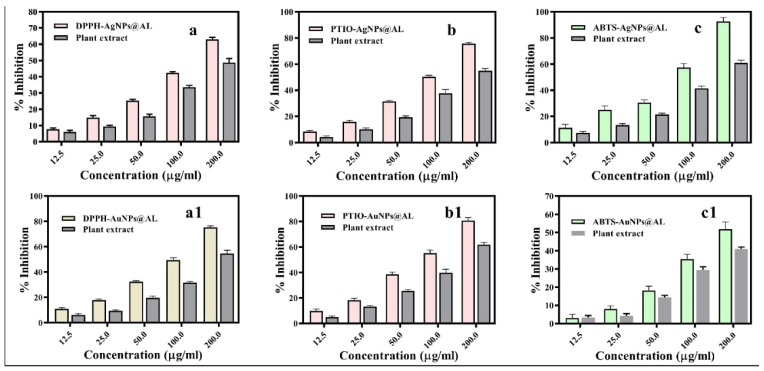
(**a**,**a1**) DPPH radical scavenging assay, (**b**,**b1**) PTIO scavenging activity and (**c**,**c1**) ABTS radical neutralization activity of Ag and AuNP@AL. The values are significantly different (*p* < 0.05).

Using a concentration-dependent test, the antioxidant potential of the Ag and AuNP@AL was evaluated by scavenging the DPPH free radical. In comparison to plant extract, the AgNP@AL exhibited significantly higher (*p* < 0.05) inhibition, as illustrated in Figure 10a. Across various concentrations of the AgNP@AL (12.5, 25, 50, 100, and 200 µg/mL), a significant enhancement in the scavenging effect was noted, displaying percentages of 7.78, 14.88, 25.2, 42.4, and 62.9%, respectively. For additional quantification and comparative assessment of the effectiveness of Ag NPs, the half-maximal inhibitory concentration (IC_50_) values were determined. In this investigation, the IC_50_ value for the AgNP@AL was computed to be 144.90 µg/mL. Similarly, in a dose-dependent assay, the AuNP@AL exhibited significantly higher potential in neutralizing DPPH, demonstrating a maximum scavenging capacity of 75.2% at 200 µg/mL and an IC_50_ value of 115.9 µg/mL (Figure 10(a1)).

It is suggested that the antioxidant activity of biogenic AuNPs might arise from their capability to transfer hydrogen or an electron to the free radical (DPPH•), thereby transforming it into stable DPPH-H [86]. In the process of transferring electrons to DPPH radicals, the metal atoms in the Ag and AuNPs undergo oxidation. Subsequently, they acquire electrons from the capped biomolecules, leading to their reduction. This cycle continues, allowing the Ag and AuNPs to effectively reduce the targeted radicals. The enhanced DPPH scavenging capability of the NPs@AL may result from a synergistic effect of both the Ag and Au components, as well as the biomolecules acting as capping agents in the synthesized NPs@AL.

#### 2.2.3. PTIO Radical Scavenging Assay

To further confirm the antioxidant capabilities of the synthesized NPs, a 2-phenyl-4,4,5,5-tetramethylimidazoline-1-oxyl 3-oxide radical (PTIO•) trapping assay was employed for the study. Various concentrations of the Ag and AuNP@AL and plant extract were employed, and their scavenging activities were recorded and analyzed. The results revealed that at a concentration of 200 µg/mL, the AgNP@AL exhibited significantly higher (*p* < 0.05) inhibition scavenging activity of 75.63%, with an IC_50_ value of 116.36 µg/mL (Figure 10b). Similarly, the significantly highest antioxidant capacity of the AuNP@AL against PTIO, reaching 75.63%, was noted at a concentration of 200 µg/mL, with an IC_50_ value of 103.54 µg/mL (Figure 10(b1)). The increased PTIO antioxidant capacity of the AuNPs facilitated by *A. Laeve* leaves, in comparison to the plant extract alone, suggests a synergistic effect arising from both the Au and the capped biomolecules. The antioxidant activity increased with rising concentrations of the AgNP@AL, AuNP@AL, and the plant extract, demonstrating a dose-dependent behavior.

#### 2.2.4. ABTS Radical Scavenging Assay

The ABTS radical method is commonly utilized for the determination of free radical concentrations, offering valuable insights into the mechanisms of both electron and hydrogen transfer involved in cationic free radical scavenging activity. A range of concentrations of the NPs@AL (12.5, 25, 50, 100, and 200 µg/mL) and plant crude extract were utilized, demonstrating substantial scavenging activity against cationic free radicals. The ABTS^•+^ scavenging activity of both the AgNP@AL and the plant crude extract demonstrated a dose-dependent rise. Particularly, at concentrations of 200 µg/mL, the AgNP@AL exhibited significantly higher (*p* < 0.05) inhibition scavenging activity, reaching approximately 92.63%, with an IC_50_ value of 95.39 µg/mL (Figure 10c). Yet, the AuNP@AL exhibited a moderate capacity to neutralize ABTS, demonstrating a significantly maximum activity of 53% at the highest dosage applied, along with an IC_50_ value of 180.85 µg/mL (Figure 10(c1)). Results from the antioxidant assays suggest that the synthesized NPs@AL in this study demonstrate notable effectiveness in scavenging various radicals, indicating their potential as a source of safe nanomedicine and antioxidants. However, further investigation is necessary to validate their practical application in in vitro models and to clarify their safety profile regarding cellular toxicity.

### 2.3. Photocatalytic Activity of AuNP@AL

**Figure 11:**
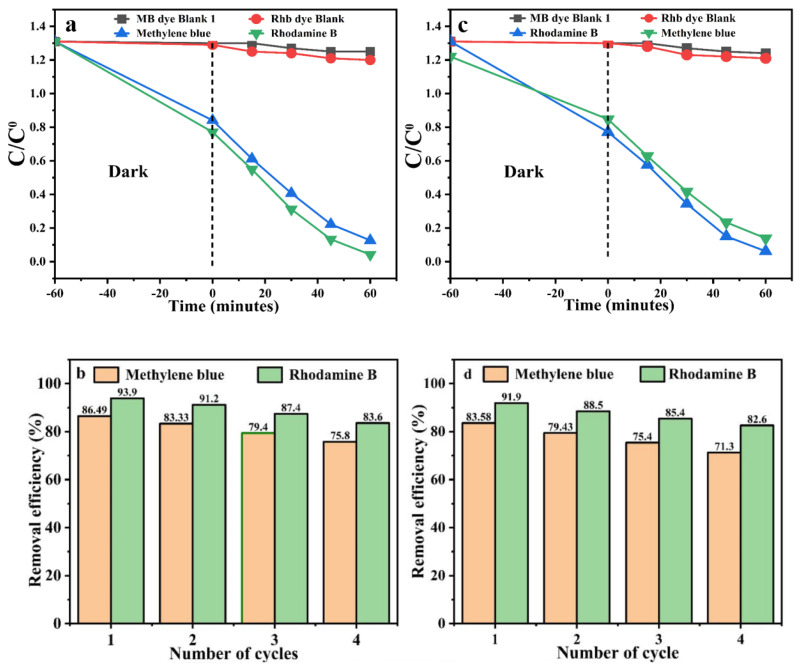
Photocatalytic performance of the biologically synthesized Ag and AuNP@AL. (**a**) Plots of ln(C/C0) versus time for the photocatalytic reduction of methylene blue (MB) and rhodamine B. (**b**) Cyclability performance of the catalyst in the catalytic removal of MB and rhodamine B dyes. (**c**,**d**) Photocatalytic activity and cyclability performance of the AuNP@AL against the model pollutants.

The catalytic performance of the NPs@AL was assessed in the photocatalytic degradation of environmental pollutants, methylene blue and rhodamine B (20 mg/L), under visible light irradiation. Monitoring the reduction in absorbance data at 668 nm for methylene blue and 553 nm for rhodamine B using a UV–Vis spectrophotometer provided insights into the NPs’ photocatalytic performance. The AgNP@AL exhibited higher photocatalytic performance, achieving the removal of 86.49% of methylene blue and 93.9% of rhodamine B from the solution (Figure 11a). The stability of the AgNPs was assessed through a recyclability test, where the NP catalyst was reused multiple times after each photocatalytic reaction. In the first cycle, the AgNP@AL exhibited robust stability, removing 86.49% of methylene blue, followed by 83.33%, 79.4%, and 75.8% in the second, third, and fourth cycles, respectively. Similarly, the AgNPs maintained stability against rhodamine B, degrading 93.9% in the first cycle and 91.2%, 87.4%, and 83.6% in the subsequent second, third, and fourth cycles under identical conditions (Figure 11b). These results imply that the AgNPs in this study exhibit higher potential for catalyzing a photocatalytic reaction and remain effective even after multiple recycling, consistent with prior research [82]. The AuNPs demonstrated efficient photocatalytic performance, eliminating 83.58% of methylene blue (MB) and 91.9% of rhodamine B (RhB) dye from the system within 60 min (Figure 11c). The stability of the synthesized AuNP@AL was calculated through a recyclability test involving the reuse of the AuNP photocatalyst after each photocatalytic reaction, repeated four times. The AuNPs demonstrated greater stability, achieving a removal efficiency of 83.58% in the first cycle, followed by 79.43%, 75.4%, and 71.3% degradation of methylene blue in the second, third, and fourth cycles, respectively. Similarly, the AuNP photocatalyst exhibited remarkable stability against Rhodamine B (RhB), with degradation rates of 91.9% in the first cycle and 88.5%, 85.4%, and 82.6% in the second, third, and fourth cycles under the same experimental conditions (Figure 11d). These findings suggest that the AgNPs in this investigation possess notable potential for catalyzing photocatalytic reactions and maintain their effectiveness even through multiple recycling, aligning with previous research [87].

## 3. Materials and Methods

### 3.1. Preparation of Plant Extract

*A. Laeve* plant materials were thoroughly washed with tap water to remove the dust, shade-dried for 10 days, and then ground to a fine powder using a blender. Subsequently, 100 g of the powdered material was suspended in 5 L of methanol in a sealed glass container and kept at room temperature for 10 days. A rotary evaporator was used to concentrate the filtered methanolic extract, producing 7.5 g methanolic crude extract that was kept refrigerated at 4 °C in a sealed container.

### 3.2. Synthesis of AgNPs@AL

The process outlined in our previous study was followed in the green synthesis of AgNPs [88,89]. In a 100 mL beaker, 5 mL of AgNO_3_ salt solution (0.002 M) in deionized water (DW) was introduced and stirred continuously for 5 min. Subsequently, 5 mL of *A. Laeve* crude extract (20 mg/mL in DW), with a pH adjusted to approximately 8 (with 5% of NaOH solution), was added dropwise. After 50 min of stirring the reaction mixture, it underwent three washing cycles and a centrifugation process (6000× *g* for 15 min). The NPs that were collected were then freeze-dried in order to allow them to be used later.

### 3.3. Synthesis of AuNP@AL

The green synthesis of AuNPs was carried out using a modified procedure from the literature [88,89]. In a standard experimental setup, 5 mL of the crude extract containing 20 mg/mL was added to a 2 mM HAuCl_4_^–^ solution (5 mL) in a beaker, and the mixture was stirred for 40 min at room temperature. The resulting colloidal suspension underwent five rounds of centrifugation and washing to obtain AuNPs. The collected pellet was freeze-dried, stored, and subsequently used for further experimental investigations.

### 3.4. Characterization of Prepared NPs

The AgNP@AL and AuNP@AL synthesized through a green method were subjected to characterization using several analytical techniques. A D/MAX-RB X-ray diffractometer from Rigaku, Japan, fitted with a Cu Kα source (λ = 1.5418 Å) running at 40 kV and 30 mA, was used to conduct an X-ray diffraction (XRD) study. A Nicolet iS50 spectrometer (Thermo Scientific, Waltham, MA, USA) was used to obtain FT-IR spectra. Scanning electron microscopy (SEM) was carried out using an S-4800 microscope (Hitachi, Tokyo, Japan) operating at 20 keV to evaluate the morphology. UV–visible spectral measurements were performed using a UV–Vis spectrophotometer equipped with an integrating sphere (T9s; Persee, Guangzhou, China). BaSO_4_ solution was used as a blank reference. Moreover, at an acceleration voltage of 200 kV, TEM and HR-TEM imaging were performed (F-20, FEI, Lexington, KY, USA). Using Al Kα radiation, an X-ray photoelectron spectrometer (ESCALAB 250Xi; Thermo, USA) was used for XPS characterization.

### 3.5. Antioxidant Assays of the Prepared NPs

#### 3.5.1. 1,1-Diphenyl-2-Picrylhydrazyl (DPPH) Assay

Various concentrations of the fabricated NPs@AL were used to examine their DPPH scavenging activity, following a procedure outlined elsewhere with minor modifications [90]. To summarize, 1 mL of the freshly prepared DPPH solution (1 mM in methanol) was mixed with 1 mL of the pre-determined quantities (12.5, 25, 50, 100, and 200 µg/mL) of the NPs@AL and plant extract (same dilutions), and then carefully vortexed. Afterwards, the mixture was kept at room temperature in the dark for half an hour. Using methanol (1 mL as blank) and DPPH (1 mL, 1 mM in methanol) as a control, the absorbance of the reaction mixtures was measured at 517 nm using a spectrophotometer. The following formula was used to determine the % inhibition of the DPPH free radical for both the plant extract and the NPs@AL:

$$DPPH\;\%\;Inhibition=\frac{Abs\;of\;control-Abs\;of\;Sample}{\;Abs\;of\;Control}\times 100\%$$

The IC_50_ values for both the extract and NPs were determined from the graph using the equation Y = mX and the linear regression coefficient.

#### 3.5.2. 2-Phenyl-4,4,5,5-Tetramethylimidazoline-1-Oxyl 3-Oxide (PTIO) Assay

The PTIO assay was carried out in accordance with the previously described method, with minor adjustments [88,91]. From each concentration (12.5, 25, 50, 100, and 200 µg/mL) of the NPs@AL and plant extract, aliquots of 200 µL were taken and combined with 800 µL of the PTIO solution (1 mM), followed by a thorough mixing. The resultant solution underwent incubation for 2 h in the dark at room temperature to prevent light-induced degradation of PTIO radicals. The absorbance of each solution was measured against the control (water plus PTIO) using a microplate reader at a wavelength of 557 nm. Finally, the percentage scavenging capacity of PTIO for each concentration of the biogenically synthesized NPs@AL and plant extract was calculated using the following formula:

$$PTIO\;\%\;Inhibition=\frac{Abs\;of\;control-Abs\;of\;Sample}{\;Abs\;of\;Control}\times 100\%$$

#### 3.5.3. The ABTS Assay

The ABTS radical scavenging activity of the NPs@AL and the crude extract was evaluated in a dose-dependent manner using the previously described method with some changes [88,92]. Accordingly, ABTS (7 mM) and potassium persulfate (2.45 mM) were combined in ethanol in order to produce an ABTS stock solution. This solution was then incubated at room temperature in the dark for 16 h. Following that, 20 µL of varying concentrations (12.5, 25, 50, 100, and 200 µg/mL) of the NPs@AL and crude extract were added to 180 µL of the ABTS working solution. To obtain an absorbance of around 0.75 ± 0.20 at 734 nm, the stock solution was diluted with methanol to produce the working solution. The solution was left undisturbed for 30 min at room temperature, and the absorbance was recorded at 734 nm using a spectrophotometer. Ethanolic ABTS solution was used as a control. The percentage inhibition for both the NPs@AL and the plant extract was calculated using the specified formula.

$$ABTS\;\%\;Inhibition=\frac{Abs\;of\;control-Abs\;of\;Sample}{\;Abs\;of\;Control}\times 100\%$$

Regression analysis was used to determine the IC_50_ value or the concentration at which 50% scavenging is achieved. A more robust capacity to neutralize DPPH, PTIO, and ABTS radicals is indicated by lower IC_50_ values.

#### 3.5.4. Antibacterial Activity

The antibacterial efficacy of the NPs@AL was examined using the agar well diffusion method using *Escherichia coli* (*E. coli*) and *Staphylococcus aureus* (*S. aureus*) as representative pathogens. The bacterial strains were cultured in LB media for 24 h. Sterilized Petri plates were filled with approximately 20 mL of molten nutrient agar media and left to solidify, followed by the application of prepared doses of the NPs@AL (80, 40, 20, 10 μg/mL) to the agar plates while bacterial cultures were incubated for 24 h at 37 °C. Zones of inhibition were measured in millimeters to evaluate the antibacterial activity of the Ag and AuNPs.

#### 3.5.5. Photocatalytic Activity of NPs@AL

The photocatalytic potential of the NPs@AL was evaluated by the photocatalytic degradation of methylene blue (MB) and rhodamine B as experimental dyes. In this experiment, a 400 W Xenon lamp with a UV cut-off filter (λ > 420 nm) was used as the radiation source. The distance between the lamp and the suspension samples containing photocatalysts and dyes was maintained at approximately 10 cm. In summary, 30 mg of the NPs@AL underwent sonication in 30 mL solutions containing methylene blue and rhodamine B dyes. To achieve full saturation of the catalyst and establish adsorption–desorption equilibrium, the reaction mixtures containing the dyes and the photocatalyst were stirred in the dark for 60 min before irradiation. During photocatalytic reactions, aliquots (2 mL) were taken at 15 min intervals to investigate the temporal impact by measuring the absorbance of both methylene blue and rhodamine B dyes. These aliquots were subsequently subjected to centrifugation to eliminate any residual photocatalyst. By tracking the ratios (C/C_0_) of the dye substrates for methylene blue and rhodamine B, where C_0_ denoted the starting concentration and C the concentration at a given time, the degradation rate was determined. This evaluation was based on absorbance measurements made with a UV–Vis spectrophotometer at 668 nm for methylene blue and 553 nm for rhodamine B.

### 3.6. Statistical Analysis

The measurements were performed in triplicate and subjected to an analysis of variance (ANOVA) using the SPSS 27.0 statistical analysis system. Statistical analysis was conducted using Duncan’s multiple range test, with a significance level of *p* < 0.05 to determine any significant differences between the means.

## 4. Conclusions

In conclusion, this scientific contribution highlights the successful fabrication of Ag and Au NPs through a green protocol. The methanol extract of *A. Laeve* crude served as both the reducing and capping agent, eliminating the requirement for additional supportive chemicals. Our results indicate that under the given experimental conditions, NPs with a well-dispersed and nearly spherical morphology were generated. The produced NPs@AL were employed as agents with antibacterial, antioxidant, and photocatalytic properties. The NPs@AL demonstrated notable efficacy in inhibiting the growth of tested bacterial strains, scavenging various radicals, and mitigating environmental pollutants such as methylene blue (MB) and rhodamine B. The biological applications, encompassing antibacterial and antioxidant activities, suggest that the green method-synthesized NPs@AL may have potential utility in treating bacterial infections and addressing medical conditions associated with oxidative stress. However, a detailed investigation is necessary to evaluate the bioavailability and biocompatibility of these NPs in both in vitro and in vivo models. These findings additionally suggest that the NPs synthesized through plant-mediated methods possess significant potential for future applications in catalysis and biomedical sciences.

## Data Availability

Data are contained within the article.
